# Vitamin D Supplementation and Blood Pressure in Children and Adolescents: A Systematic Review and Meta-Analysis

**DOI:** 10.3390/nu12041163

**Published:** 2020-04-22

**Authors:** Myriam Abboud

**Affiliations:** Department of Health, CNHS, Zayed University, Dubai P.O. Box 19282, UAE; myriam.abboud@zu.ac.ae

**Keywords:** vitamin D, blood pressure, children, adolescents, systematic review, meta-analysis

## Abstract

Suboptimal vitamin D status is associated with elevated blood pressure (BP) in children and adolescents. Whether vitamin D supplementation reduces BP remains unclear. To systematically review whether vitamin D supplementation reduces BP in children and adolescents, we conducted a literature review according to the PRISMA statement. We included vitamin-D supplementation human interventions studies that reported on BP as an outcome. We searched PUBMED, MEDLINE, CINAHL, EMBASE, the Cochrane Library, and the clinical trials website. We also hand searched the references of the included articles and previous reviews of vitamin D therapy. No language or time restrictions were applied. We extracted data on population characteristics, baseline and endline vitamin D and BP values, and assessed the risk of bias of the included studies. We performed a narrative review of the findings, conducted a meta-analysis when possible, and performed sensitivity analyses to test the robustness of our results. We assessed the overall quality of the evidence produced in the meta-analysis. We included eight studies in our review and five studies in the meta-analysis, none of which included hypertensive only participants. The risk of bias was variable. In non-randomized studies, no effect of vitamin D supplementation was seen on systolic BP (SBP) (mean difference: 0.39 (95% confidence interval (CI): −0.9; 1.68) mmHg; *p =* 0.55; I^2^ = 0%). Only a significant decrease in diastolic BP (DBP) (mean difference: −1.87 (95% CI: −3.02; −0.72) mmHg; *p =* 0.001; I^2^ = 0%) was noted. Both analyses had a low quality of evidence. In randomized controlled trials (RCTs), no effect was noted on SBP (mean difference: −2.04 (95% CI: −5.12; 1.04) mmHg; *p =* 0.19; I^2^ = 71%) nor DBP (mean difference: 0.01 (95% CI: −1.09; 1.12) mmHg; *p =* 0.98; I^2^ = 0%). The final quality of evidence ranged between low and moderate. Sensitivity analyses did not affect the results. Vitamin D supplementation was found to be ineffective in lowering SBP and DBP in children and adolescents.

## 1. Introduction

Vitamin D, recently coined as the D hormone, is a pleiotropic steroid hormone that has multiple biological effects. Most notably, it plays an integral function in the regulation of calcium and phosphorus homeostasis, and thus has a vital role in bone health. Emerging evidence suggests further extra-skeletal physiological actions, but clinical consequences are still debatable [1,2,3,4].

Hypovitaminosis D, or low serum levels of 25-hydroxyvitamin D (25OHD), is widespread in both adults and children around the world [5,6]. The most common determinants of deficiency include limited sunlight exposure, diseases that cause malabsorption (i.e., celiac disease, cystic fibrosis), diet, obesity, and altered metabolism secondary to some medications [4,7].

Hypertension, or elevated blood pressure (BP), is a well-recognized risk factor for both cardiovascular and renal diseases in addition to a vast array of diseases contributing significantly to mortality [8,9]. Specifically, childhood hypertension poses a considerable public health challenge [10], and is associated with essential hypertension in adults and detrimental cardiovascular events [9,11]. Recent pooled estimates suggest the global prevalence of childhood hypertension has reached 4%, and that it is generally more common in adolescents undergoing puberty and children who are overweight or obese. Furthermore, an upward trend in childhood hypertension has been observed during the past two decades, with a relative rate increase of 75% to 79% from 2000 to 2015. This increase in prevalence is expected to persist in the future [10].

Among adults, a wealth of observational data has demonstrated a relationship between low serum levels of 25OHD and hypertension [12,13,14], although no evidence of a clinically significant reduction in BP as a result of vitamin D supplementation has been observed [14].

Among children and adolescents, Dolinsky et al. systematically reviewed the association between vitamin D and BP [15]. The majority of included observational studies found an inverse association between 25OHD and systolic blood pressure (SBP) [14]. Likewise, obese children with low levels of vitamin D showed increased odds for hypertension, even after adjusting for body mass index and body fat [16]. Such observations are supported by biologically plausible mechanisms that could mediate an effect of vitamin D on BP, such as the presence of vitamin D receptors on endothelial cells, smooth muscle cells, and myocytes [17], or the beneficial effect of vitamin D in improving endothelial function, reducing the production of proinflammatory cytokines, regulating the activity of the renin–angiotensin–aldosterone system and preventing secondary hyperparathyroidism [14,18,19,20]. Nevertheless, human interventions studies have produced conflicting evidence on the antihypertensive effect of supplementation with vitamin D, where no change in SBP or diastolic blood pressure (DBP) was recorded even when 25OHD increased upon supplementation [21]. The fact that it is unlikely that vitamin D supplements are beneficial for improving bone density in healthy children and adolescents led us to conduct this study and explore the effect of these supplements on BP in children and adolescents.

Preventing vitamin D deficiency or correcting it through supplementation could be of public health relevance, especially since it is an inexpensive intervention already used in children and adolescents to improve bone health. This study aims to systematically review the literature on the relationship between vitamin D supplementation and BP in children and adolescents, and meta-analyze available data.

## 2. Methods

### 2.1. Review Design

The review was conducted according to the Preferred Reporting Items for Systematic Reviews and Meta-Analyses (PRISMA) statement. We conducted the systematic review following a predefined protocol that was submitted to the International Prospective Register of Systematic Reviews (PROSPERO). Ethical approval was not required for the current study. We included human interventions studies that reported on BP (systolic or diastolic) as an outcome, and supplementation with vitamin D. We also included observational studies that reported BP (systolic or diastolic) as an outcome, and vitamin D deficiency as an exposure. We searched the following databases: PUBMED, MEDLINE, CINAHL, EMBASE, the Cochrane Library, and the clinical trials website [22].

We also hand searched the references of the included articles and previous reviews of vitamin D status and therapy in children and adolescents as identified by the search. No language or time restrictions were applied to eligible reports. For this review, only studies reporting on vitamin D supplementation and BP were considered.

### 2.2. Search Strategy

We considered three key concepts: (1) vitamin D, (2) blood pressure, and (3) children and adolescents. For each of the three concepts, we mapped Medical Subject Headings (MeSH) and keywords. Search terms included but were not limited to: vitamin D, cholecalciferol, ergocalciferol or calcidol, combined with blood pressure or hypertension, and pediatric, child, adolescent, youth or teenage. The search period spanned 1 January 1966, through 17 January 2020. The electronic search strategy was validated by a medical information specialist and is described in the Supplementary Materials.

### 2.3. Study Selection

We considered cross-sectional, case-control, retrospective, or prospective cohort studies; and randomized, non-randomized, controlled, or uncontrolled clinical trials including children and adolescents as defined by the studies (e.g., aged less than 18 years). For this review, we included studies evaluating the effect of vitamin D supplementation on BP reduction. We specifically considered interventions such as vitamin D3 (cholecalciferol) or other forms of supplementation, including vitamin D-fortified milk with a duration of at least 4 weeks to ensure that the intervention had sufficient time to produce an effect. The main outcome for controlled trials was the difference in measured BP (SBP, DBP or mean arterial pressure) readings between control and intervention groups, and the main outcome for non-controlled trials was the change in measured BP readings from baseline through follow-up.

Studies solely including adult patients as defined by the studies (e.g., aged 18 years and above), studies conducted on pregnant women, cord blood, neonates and infants, studies focused on participants with diseases (e.g., inborn errors of metabolism, cancer, transplant, seizures, chronic kidney disease, dialysis, liver disease, parathyroid abnormality, and vitamin D-dependent rickets types 1 and 2), studies focused on participants taking medications known to interfere with vitamin D metabolism (e.g., phenytoin, phenobarbital, carbamazepine, and rifampin), and studies evaluating the association between hypovitaminosis (deficiency or insufficiency of vitamin D status) or hypervitaminosis D and BP were excluded.

The author screened titles and/or abstracts retrieved by the search and identified studies that potentially met the inclusion criteria outlined above. The full texts of potentially eligible studies were retrieved and assessed for eligibility.

### 2.4. Data Extraction

The author extracted data from eligible studies using a data extraction form. For all eligible records, the author recorded the characteristics of the study, details of the population included, the intervention applied and its comparator, as well the main findings and adjustments to the analyses, where applicable.

Serum 25OHD was converted to nanomoles per liter (nmol/L) if it was reported as nanograms per milliliter (ng/mL) by multiplying by a factor of 2.496.

### 2.5. Risk of Bias Assessment

The author assessed each included study for risk for bias. For randomized controlled trials, the Cochrane criteria [23] (sequence generation, allocation concealment, blinding of participants and outcome assessors, incomplete outcome data, and selective outcome reporting) were used. For non-randomized studies, a modified version of the Cochrane Risk of Bias tool [24] (eligibility criteria, measurement of exposure and outcome, control confounding, and follow-up) was used. Each potential source of bias was graded as low, high, or unclear risk.

The overall quality of the evidence produced in the meta-analysis was evaluated according to the Grading of Recommendations Assessment, Development, and Evaluation (GRADE) criteria (high risk of bias, imprecision, indirectness, heterogeneity, and publication bias) with the use of GRADEpro tool (Evidence Prime Inc., McMaster University, Hamilton, ON, Canada).

### 2.6. Data Synthesis

Using RevMan version 5.3 (The Cochrane Collaboration, The Nordic Cochrane Centre), we performed standard meta-analysis comparing vitamin D supplementation with no supplementation according to a random-effects model. We reported on the results of the meta-analysis as the weighted mean difference with 95% confidence intervals. Meta-analysis was performed when participants, treatments and the outcomes were similar enough to allow pooling. When the trial consisted of two groups, we pooled the two arms of interventions using the formula for combining two arms from the Cochrane handbook (chapter 16.5.4, table 7.7.a.) [23]. When a meta-analysis was not possible, we performed a narrative review of the findings. We used the I^2^ statistic to assess heterogeneity among different studies in each comparison. In cases of moderate to substantial heterogeneity, with I^2^ values greater than 50%, we explored and reported on the potential causes. Also, we conducted sensitivity analyses in regard to having or not having vitamin D deficiency as the inclusion criterion. The sensitivity analyses were conducted to test the robustness of our results.

## 3. Results

### 3.1. Search Results

**Figure 1:**
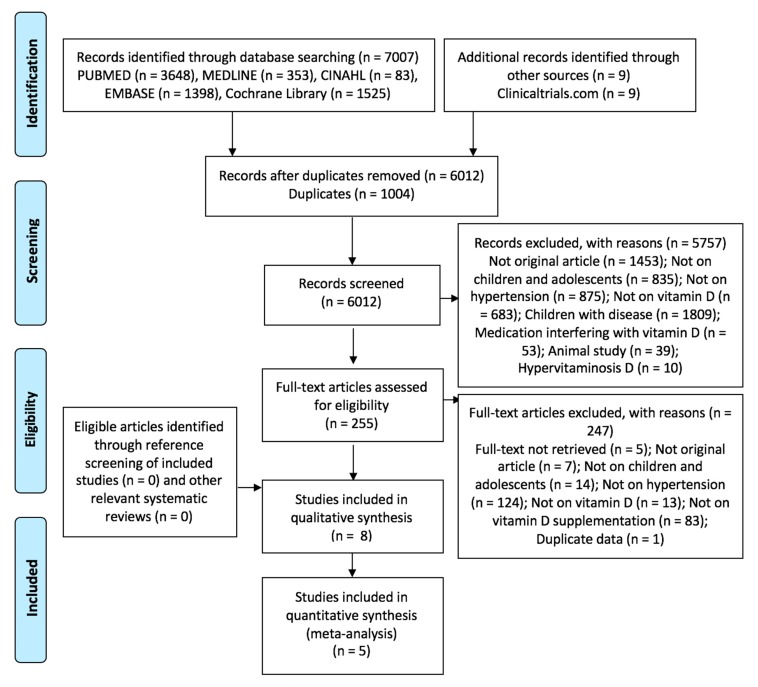
Preferred Reporting Items for Systematic Reviews and Meta-Analyses (PRISMA) diagram of study selection.

Details of the search process are presented in Figure 1. We included eight studies in the systematic review, of which five studies yielded data that could be combined in the meta-analysis.

### 3.2. Characteristics of Included Studies

The characteristics of the included studies are given in Table 1 and Table 2.

The articles included three non-randomized human interventions studies [25,26,27] and five controlled trials of vitamin D supplementation [28,29,30,31]. Three of the studies were conducted in the USA [25,26,29], two in Iran [27,30], one in Denmark [32], and one in each of Saudi Arabia [28] and the United Kingdom [31]. Participant age in the studies ranged from 4 to 18 years, and participant sample size ranged from 14 to 940. Two of the non-randomized trials [25,26] were conducted on obese adolescents, and two of them [25,27] included females exclusively. As for the RCTs, all of them were conducted on reportedly healthy individuals, except in Kelishadi et al. [30] which included participants with obesity and metabolic syndrome. In this study [30], the authors adopted a continuous value of metabolic syndrome, which was calculated as the sum of the standardized residuals (Z-scores) of the individual variables of waist circumference, high density lipoprotein cholesterol (multiplied by −1), triglycerides, fasting blood glucose, and mean arterial blood pressure based on age and gender, and whereby a higher continuous value of metabolic syndrome score indicated a less favorable metabolic profile. This score is validated in Iranian children and adolescents.

None of the studies, whether randomized or not, included only hypertensive participants. Further, in Khayyatzadeh et al. [27], Javed et al. [26], Dong et al. [29], Al Daghri et al. [28], Hauger et al. [32] and Smith et al. [31], only healthy, non-hypertensive subjects were recruited. No details regarding baseline prevalence of hypertension were provided in Ashraf et al. [25]. Finally, although Kelishadi et al. [30] included participants with metabolic syndrome, the prevalence of elevated BP at baseline was not reported. All of the studies assessed changes in SBP and DBP, except Al Daghri et al. [27], who also assessed the prevalence of elevated BP (defined as ≥90th percentile for age, sex and height), and Kelishadi et al. [30], who solely assessed mean arterial pressure.

Regarding vitamin D status, two out of the three non-randomized human interventions studies were conducted on participants with either vitamin D deficiency [25] or insufficiency [26]. Inadequate vitamin D status was not an inclusion criterion only the study by Khayyatzadeh et al. [27], although around 95% of the study population were either vitamin D deficient or insufficient. In four [28,29,30,31] out of the five included RCTs, mean baseline vitamin D was less than 50 nmol/L.

In the non-randomized human interventions studies, the interventions varied between oral vitamin D2 [25] or D3 supplementation [26,27]. The daily equivalent dose ranged from 3279 IU [26] to 7143 IU [25,27], and the intervention period ranged from eight weeks [25] to three months [26]. In all included RCTs, the interventions consisted of vitamin D3 supplementation, except in the study by Al Daghri et al. [28], which also included another treatment arm consisting of supplementation through vitamin D-fortified milk. The duration of supplementation ranged from 12 weeks [29] to six months [28], with a daily equivalent dose ranging from 80 IU [28] to 42,857 IU [30].

### 3.3. Results of Included Studies

Table 3 and Table 4 describe the findings from the included studies.

All non-randomized human intervention studies reported a significant increase in 25OHD after supplementation. No significant association was reported between 25OHD and either SBP or DBP in Ashraf et al. [25] and Javed et al. [26]. Only Khayyatzadehet al. [27] reported an overall significant reduction in DBP (SD) from 62.3 (13.4) to 60.0 (12.9) mmHg, which was also noted in all of the deficient, insufficient and sufficient at baseline subcategories.

As for the RCTs, none of the studies reported significant changes in either SBP or DBP over time, in intra- or inter-group comparisons, except Al Daghri et al. [28]. In this study, a significant change in BP was noted; the authors reported a significant decrease in SBP over time in all the study groups, including the control group, with a significant inter-group difference in favor of the vitamin D tablet group. Also, Al Daghri et al. reported a significant decrease in DBP over time in both the vitamin D tablet and control groups [28]. This was a significant inter-group difference in favor of the control group. Similarly, the prevalence of elevated BP decreased significantly in the vitamin D tablet and control groups, from 38.9% to 25%, and from 34.9% to 24.7%, respectively.

### 3.4. Assessment of Risk of Bias

**Figure 2:**
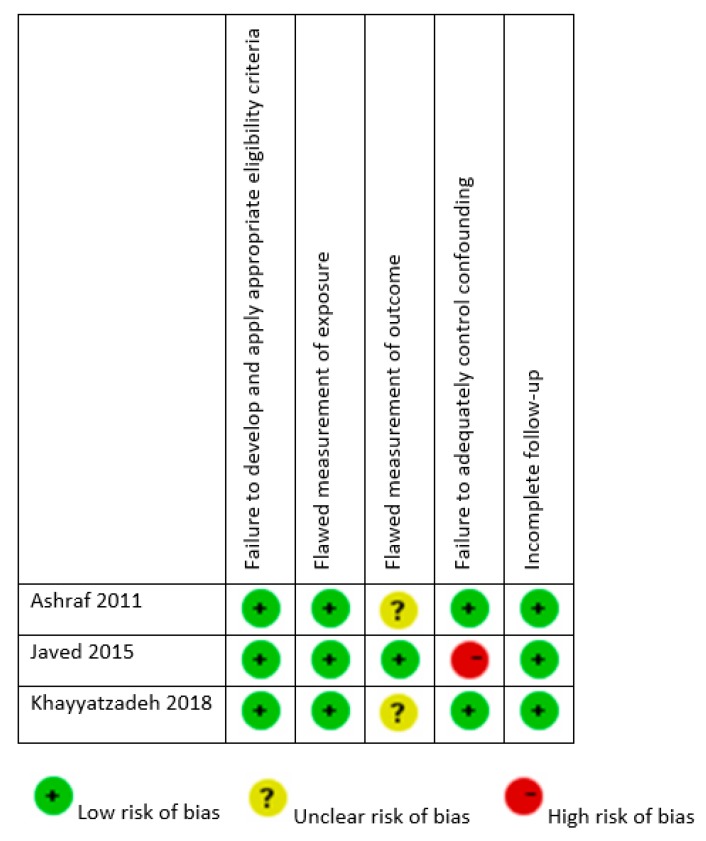
Risk of bias of non-randomized human interventions studies.

The assessment of risk of bias of non-randomized human interventions studies is presented in Figure 2. The quality of included studies was generally high. Ashraf et al. [25] and Khayyatzadeh et al. [27] provided results adjusted to potential confounders. Interestingly, none of the included studies, except that of Javed et al. [26], provided a detailed description of BP measurement.

**Figure 3:**
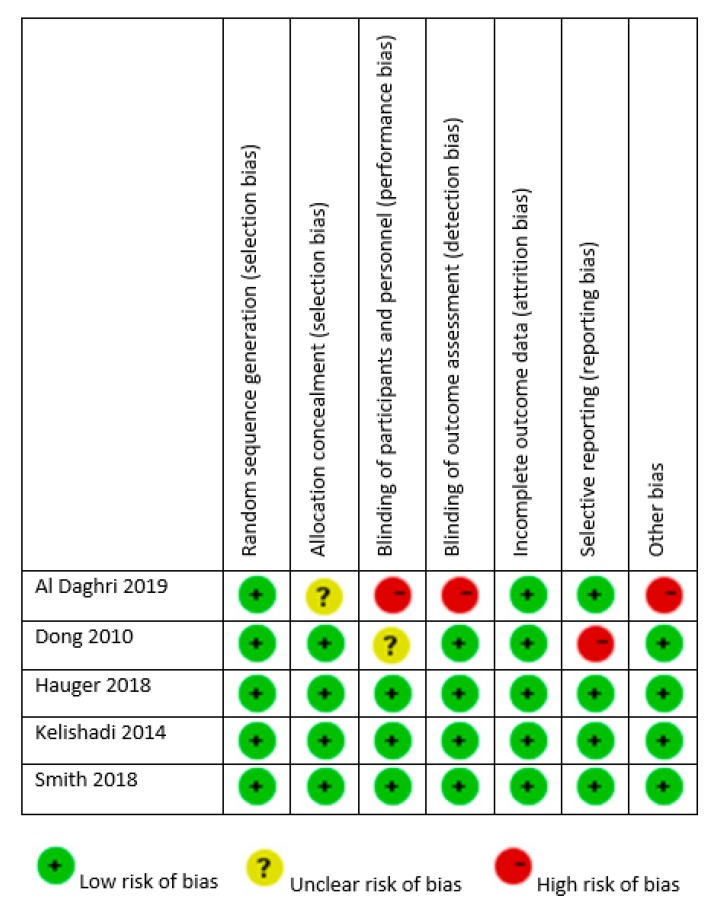
Risk of bias of randomized controlled trials.

The quality of the design and reporting of RCTs was variable, as presented in Figure 3. All studies reported random allocation, but there was insufficient detail given to ascertain adequate allocation concealment in another two studies (those by Dong et al. [29] and Al Daghri et al. [28]). All studies mentioned withdrawals and reasons for withdrawal along with dropout numbers. All trials had adequate blinding of participants and personnel, except those by Dong et al. [29] and Al Daghri et al. [28], and all reported blinding of outcome assessment. Finally, in the study by Dong et al., endline BP values were not presented [21].

### 3.5. Results of the Meta-Analyses

The studies by Javed et al. [26] and Dong et al. [29] were not included in the meta-analysis, as they did not report on endline values of SBP and DBP. Moreover, for the study by Al Daghri et al. [28], only the values of the tablet vs. control arms were included in the meta-analysis. Finally, for the studies by Hauger et al. [32] and Smith et al. [31], the two arms of the interventions were pooled and compared with the control group.

**Figure 4:**
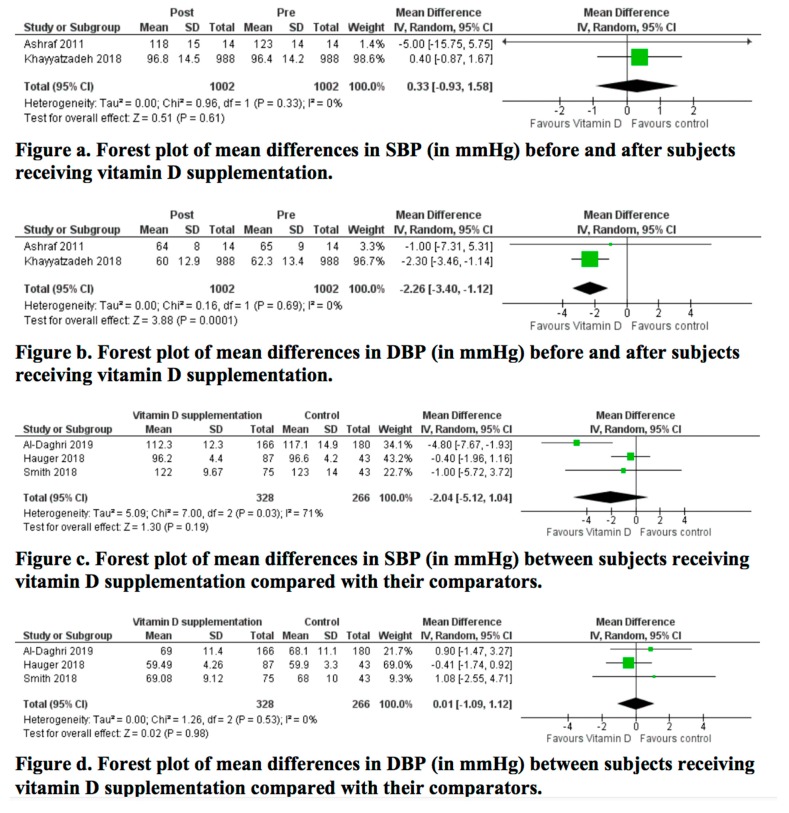
Meta-analysis of effects of vitamin D supplementation on SBP and DBP. Mean differences for each study are represented by squares, and 95% CIs are represented by the lines through the squares. The pooled mean differences are represented by diamonds. Between-study heterogeneity was assessed with the use of the I2 statistic. SBP: systolic blood pressure; DBP: diastolic blood pressure.

Forest plots for the mean difference in SBP and DBP are presented in Figure 4. Meta-analysis of the change in SBP between pre- and post-supplementation with vitamin D revealed no statistically significant difference (mean difference: 0.39 (95% CI: −0.9 to 1.68) mm Hg; *p =* 0.55; I^2^ = 0%). Regarding DBP, the meta-analysis revealed a statistically significant decrease post-vitamin D supplementation (mean difference: −1.87 (95% CI: −3.02 to −0.72) mm Hg; *p =* 0.001; I^2^ = 0%). Both analyses had a low quality of evidence as seen in the Table S1. As for the RCTs, the meta-analyses revealed no statistically significant difference in SBP (mean difference: −2.04 (95% CI: −5.12 to 1.04) mm Hg; *p =* 0.19; I^2^ = 71%) nor DBP (mean difference: 0.01 (95% CI, −1.09 to 1.12) mm Hg; *p =* 0.98; I^2^ = 0%).

The final quality of evidence of RCTs ranged between low and moderate for SBP and DBP, respectively (Table S1). Sensitivity analyses, available in Figure S1 (a,b,c,d), did not affect the results. This was also confirmed in the sensitivity analysis of the quality evidence as outlined in Table S2.

## 4. Discussion

To date, conflicting evidence exists about the effects of vitamin D supplementation on improving cardiometabolic outcomes and specifically decreasing BP. In our review and meta-analysis, we found no evidence of BP reduction in children and adolescents through supplementation with vitamin D, except for a significant decrease in DBP of about 2 mmHg in non-randomized human intervention studies. Our findings were consistent, as no change in BP was noted in children and adolescents who were either healthy [29,31,32], obese [25,26] or diagnosed with metabolic syndrome [30]. Also, our results were robust in the sensitivity analyses (Figure S1 (a,b,c,d)).

Results from the randomized studies showed no significant effect on SBP nor DBP in normotensive participants, despite correcting deficient or insufficient vitamin D baseline levels. These findings are in line with the most recent meta-analysis in children and adolescents, which failed to find any effect of vitamin D supplementation on SBP and DBP [33]. Also, this is in line with results of previous systematic reviews and meta-analyses of trials among non-hypertensive adults [34,35,36]. While some evidence exists to underpin the modest BP-lowering effect of vitamin D in adult patients who are either hypertensive [37] or with pre-existing cardiometabolic disease [38], this remains unclear [34], and we could not ascertain this issue since none of the studies included solely hypertensive patients or those suffering from cardiometabolic disease.

The optimum serum 25OHD levels in children and adolescents associated with both skeletal and extraskeletal health outcomes, and the supplementation doses required to achieve it, remain debatable [2]. Current recommended vitamin D supplementation doses for children and adolescents vary mainly between 400 and 600 IU/day [39,40,41,42,43], and all of the included studies, except Hauger et al. [31] and Smith et al. [31] in one of their intervention arms, employed doses exceeding this range. Further, all of the non-randomized human intervention studies provided doses exceeding the 2011 Institute of Medicine (IOM) [39] tolerable upper limits (UL) of daily vitamin D supplementation (2500 IU/d in children aged four to eight years and 3000 IU in older children and adolescents). Yet, all of these studies did not find a beneficial effect, even when doses doubled the UL, such as in Ashraf et al. [25] and Khayyatzadeh et al. [27], or when a weekly mega-dose of 300,000 IU (greatly exceeding the IOM UL) was administered, as in Kelishadi et al. [30]. Given the risk of hypercalcemia, and in light of the absence of any significant BP decrease as found by our study and elsewhere [33], extremely cautious provision of vitamin D doses exceeding the UL in children and adolescents should be exercised.

Existing observational data [15] suggest an inverse association between low vitamin D status and cardiometabolic health, including SBP. Thus, correcting suboptimal vitamin D status was suggested as a means to improve BP. Our analysis does not support this suggestion. Two of the non-randomized studies [25,27] included vitamin D-insufficient participants (baseline 25OHD levels below the recommended IOM level of 50 nmol/L) and both studies reported a non-significant change in BP. Similarly, in the randomized studies by Hauger et al. [33] and Smith et al. [31] including healthy and predominantly normal-weighted children and adolescents, despite a significant improvement in serum 25OHD levels in the intervention groups paralleled with a significant decline in the placebo group, BP remained unchanged across all groups. This is consistent with results noted among adults [44]. This also indicated that the seasonal fluctuation in vitamin D concentration may not compromise BP in normal-weighted individuals. It also raises the question on the feasibility of winter vitamin D supplementation on cardiometabolic health in this group. This insignificant change in BP was also recently reported by Abboud et al. [45], showing no effect of vitamin D status at baseline or of vitamin D supplementation on changes in SBP or DBP.

On the other hand, the results presented by Al Daghri et al. [28] were interesting. They showed that in patients with components of metabolic syndrome, a correction of baseline insufficient 25OHD levels (resulting in significantly higher levels than the control group) exhibited a significant decrease in SBP and DBP, as well as the prevalence of elevated BP. This could suggest a potential role of vitamin D supplementation in lowering cardiometabolic markers when elevated at baseline. Hence, vitamin D should be taken in consideration when planning treatment.

Numerous additional variables govern our interpretation of children studies involving vitamin D supplementation [2]. Among these factors is the frequency of supplementation. All of the RCTs, except Kelishadi et al. [30] and Smith et al. [31], employed regular small doses of vitamin D supplementation. Daily supplementation may have different biological effects [46] in comparison with intermittent supplementation (monthly, weekly or lesser frequency). Another aspect that could affect the interpretation of our results are the genetic variants for the causal effect of vitamin D on BP; this assessment was beyond the scope of our investigation. Ethnicity and genetic differences between populations are important determinants in vitamin D metabolism [2]. In a systematic review and Mendelian randomization analysis using published data, Kunutsor et al. [38] found that particular genetic variants (vitamin D single nucleotide polymorphisms (SNPs)) had nominally significant associations with both SBP and DBP. Additional evidence from genetic data coupled with clinical trial data is needed to identify selected subgroups who could benefit to a greater extent from vitamin D supplementation [38].

Our analysis has numerous strengths. We followed a systematic approach in our search and analysis and employed sensitivity analyses to assess the robustness of our results. In general, the risk of bias of the included studies was low.

However, our analysis had a few limitations. First, the number of identified studies was limited, with sample sizes smaller than 1000, which might have hindered our ability to detect a statistically significant effect on BP. Second, we were limited to study-level rather than individual-level data, which would have been more accurate than the overall mean change. Third, we did not contact the authors of the three articles where endline SBP and DBP values were not reported [26,29,30], and accordingly, these articles were not included in the meta-analysis. Fourth, all of the studies, except Kelishadi et al. [30], have specifically targeted patients who were normotensive at baseline. It is plausible that normal BP is more likely to remain unchanged with any intervention, including vitamin D correction. There were some variabilities among the studies, which complicates the comparisons between included studies and the interpretation of our results, especially in the methods used to assess 25OHD and BP, doses analyzed, frequency of administration and confounders adjusted for. Importantly, studies did not adjust for sun exposure, physical activity levels, and socio-economic status, all of which might modulate the effects of vitamin D interventions [2]. Finally, it was not possible to do the screening, selection of studies, data extraction, quality assessment and grading of the meta-analysis in duplicate.

## 5. Conclusions

In conclusion, the evidence presented in this review and analysis do not support the use of vitamin D supplementation in clinical practice as a BP-lowering agent in healthy or obese children and adolescents who are normotensive. Our results remain to be interpreted with caution given the limited number of included studies, specifically RCTs, as well as their relatively small sample size. We could not find any study assessing the effect of vitamin D supplementation on participants who were hypertensive or with cardiometabolic disease. Since the direct effect of vitamin D could be more potent when subjects have baseline metabolic disturbances, this remains to be explored in future adequately powered, high-quality RCTs.

## Figures and Tables

**Table 1:** Characteristics of non-randomized human interventions studies.

Author, Year	Study Design	Geographic Setting	Study Population	Age (Years) and Gender	Intervention	Duration	Daily Dose Equivalent	Compliance
Ashraf, 2011 [25]	Before/after study	Birmingham, USA	14 obese post-menarchal female adolescents (13 African American, 1 Caucasian American), with serum 25OHD < 75 nmol/L Mean BMI (SD) in kg/m^2^: NR	Mean (SD) Pre-treatment: 14.9 (1.8) Post-treatment: 15.6 (1.7)	Vitamin D2 (ergocalciferol, orally, 50,000 IU), once per week	8 weeks	7142.8 IU	NR
				% Male: 0%				
Javed, 2015 [26]	Before/after study	Rochester, USA	19 obese adolescents (89.5% non-Hispanic white), non-hypertensive, with serum 25OHD < 75 nmol/L Mean BMI (SD) in kg/m^2^: deficient: 21.2 (4.4); insufficient: 20.6 (3.6)	Range: 13–18 Mean (SD): 15.8 (1.7)	Vitamin D3 (cholecalciferol, 2 pills, 50,000 IU each; Total: 100,000 IU), once per month	12 weeks	3278.7 IU	100%
				% Male: NR				
Khayyatzadeh, 2018 [27]	Before/after study	Mashhad and Sabzevar, Iran	940 healthy female adolescents, not taking medications or vitamin D supplements Mean BMI (SD) in kg/m^2^: 21.07 (4.2)	Range: 12–18 Deficient: 14.5 (1.53) Insufficient: 14.7 (1.51) Sufficient: 15.2 (1.53)	Vitamin D3 (cholecalciferol, 1 capsule, 50,000 IU), once per week	9 weeks	7142.8 IU	NR dropout rate: 4.8%
				% Male: 0%				

25OHD: 25-hydroxyvitamin D; BMI: body mass index; SD: standard deviation; NR: not reported; IU: international units.

**Table 2:** Characteristics of RCTs.

Author, Year	Study Design	Geographic Setting	Study Population	Age (Years) and Gender	Intervention	Duration	Daily Dose Equivalent	Control	Compliance
Al Daghri, 2019 [28]	Cluster RCT	Riyadh, Saudi Arabia	535 healthy children and adolescents with 25OHD < 50 nmol/L, non-hypertensive, not taking medications or vitamin supplements Mean BMI (SD) in kg/m^2^: Total: 23.0 (6.2); Tablet: 23.0 (6.2); Milk: 23.7 (5.6); C: 24.3 (6.4)	Range: 12-18 Mean (SD): Total: 14.9 (1.9) Tablet: 14.3 (1.6) Milk: 14.4 (1.5) C: 16.1 (1.9)	Tablet: n = 166; vitamin D tablet, 1000 IU daily	24 weeks	Tablet: 1000 IU Milk: 80 IU	n = 180; no intervention	Tablet: 91.1%Milk: 90.4% C: 86.7%
				% Male: 45.4%	Milk: n = 189; 200 mL of vitamin D-fortified milk, 40 IU/100 mL, daily				
Dong, 2010 [29]	Open-label, investigator-blinded RCT	Richmond, USA	44 healthy black (African American) adolescents, non-hypertensive, not taking medications or vitamin supplements Mean (SD) BMI percentile: I: 67.8 (30.9); C: 61.6 (33.4) (*p =* 0.53)	Range: 14–18 Mean (SD): Total: 16.3 (1.4) I: 16.5 (1.4)C: 16.3 (1.1) (*p =* 0.95)	n = 25; vitamin D3 (cholecalciferol), 2000 IU/day	16 weeks	2000 IU	n = 24; vitamin D3 (cholecalciferol); 400 IU/day	I: 85% C: 88% (*p =* 0.65)
				% Male: 55.5%					
Hauger, 2018 [21]	Double blind, placebo controlled RCT	Copenhagen, Denmark	119 healthy white children of European origin, not taking vitamin D supplement for ≥1 month prior to the study and not planning a winter sun vacation Normal weight: I1: 90%; I2: 92%; C: 66%	Range: 4–8 Mean (SD): Total: 6.7 (1.5) I1: 6.9 (1.5) I2: 6.7 (1.4) C: 6.5 (1.5)	I1: n = 44; 10 μg D3 tablet/day	20 weeks	I1: 400 IU I2: 800 IU	n = 43; placebo-matching tablet (0 μg D3/day)	I1: 97.6% I2: 97.6%
				% Male: 36%	I2: n = 43; 20 μg D3 tablet/day				
Kelishadi, 2014 [30]	Triple blind, placebo controlled RCT	Isfahan, Iran	43 children and adolescents with metabolic syndrome and BMI ≥3 Z-scores, not taking medication or supplementation use, free of other chronic disease Mean BMI (SD) in kg/m^2^: C: 27.81(1.04); I: 28.08 (1.06)	Range 10–16	n = 21; 300,000 IU vitamin D3, 1 capsule/week	12 weeks	42,857 IU	n = 22; placebo-matching capsule	I: 88% C: 96% from original sample
				% Male: NR					
Smith, 2018 [31]	Double blind, placebo controlled RCT	United Kingdom	102 healthy white adolescents, not taking vitamin D supplement or planning a winter sun vacation Normal weight: I1: 80%; I2: 84%; C: 79%	Range: 14–18 Mean (SD): 15.9 (1.4) I1: 16.0 (1.4) I2: 15.9 (1.5) C: 15.9 (1.4)	I1: n = 39; 10 μg D3 tablet/day I2: n = 36; 20 μg D3 tablet/day	20 weeks	I1: 400 IU I2: 800 IU	C: n = 43; placebo- matching Tablet (0 μg D3/day)	I1: 94.2% I2: 94.4%
				% Male: 43%					

RCT: randomized controlled trial; 25OHD: 25-hydroxyvitamin D; BMI: body mass index; SD: standard deviation; C: control; IU: international units; I: intervention; NR: not reported.

**Table 3:** Results of non-randomized human intervention studies.

Author, Year	Outcomes Evaluated	Mean (SD) Baseline 25OHD (nmol/L)	Mean (SD) Endline 25OHD (nmol/L)	Mean (SD) Baseline BP (mmHg)	Mean (SD) Endline BP (mmHg)	Conclusion
Ashraf, 2011 [25]	Serum 25OHD: liquid chromatography-tandem mass spectrometry	26.4 (10.9)	63.6 (30.2) (*p <* 0.001)	SBP: 123 (14) DBP: 65 (9)	SBP: 118 (15) (*p*: 0.41) DBP: 64 (8) (*p*: 0.72)	NS change in SBP and DBP
	SBP and DBP: automated blood pressure cuff appropriate for arm size (number of measurements: NR)					
Javed, 2015 [26]	Serum 25OHD: liquid chromatography-tandem mass spectrometry	55.9 (12.2)	86.9 (16.7) (*p <* 0.001)	NR	NR	NS change in SBP and DBP
	SBP and DBP: average of 2 measures by aneroid sphygmomanometer with the participant’s arm supported and positioned at the level of the heart taken after >10 minute rest					
Khayyatzadeh, 2018 [27]	Serum 25OHD: electrochemi-luminescence	Total: 23.6 (22.04) Deficient: 17.2 (9.4) Insufficient: 60.07 (8.1) Sufficient: 99.5 (22.2)	Total: 90.9 (38.6) (*p <* 0.001) Deficient: 89.1 (37.7) (*p <* 0.001) Insufficient: 99.9 (46.9) (*p <* 0.001) Sufficient: 116.1 (36.6) (*p <* 0.001)	SBP: Total: 96.4 (14.2) Deficient: 96.6 (14.2) Insufficient: 98.3 (14.3) Sufficient: 98.8 (11.2)	SBP: Total: 96.8 (14.5) (*p =* 0.63 in adjusted model) Deficient: 97.1 (14.6) (*p =* 0.48) Insufficient: 98.2 (13.1) (*p =* 0.77) Sufficient: 95.6 (14.2) (*p =* 0.05)	Significant reduction in DBP and NS change in SBP
	SBP and DBP: standard procedure (procedure not detailed)			DBP: Total: 62.3 (13.4) Deficient: 62.5 (13.05) Insufficient: 64.5 (12.8) Sufficient: 66.05 (10.4)	DBP: Total: 60.0 (12.9) (*p =* 0.03 in adjusted model) Deficient: 60.7 (13.01) (*p =* 0.005) Insufficient: 60.9 (10.5) (*p <* 0.001) Sufficient: 61.9 (12.7) (*p =* 0.002)	

SD: standard deviation; BP: blood pressure; 25OHD: 25-hydroxyvitamin D; SBP: systolic blood pressure; DBP: diastolic blood pressure; NS: not significant; NR: not reported; IU: international units.

**Table 4:** Results of RCTs.

Author, Year	Outcomes Evaluated	Mean (SD) Baseline 25OHD (nmol/L)	Mean (SD) Endline 25OHD (nmol/L)	Mean (SD) Baseline BP (mmHg)	Mean (SD) Endline BP (mmHg)	Conclusion
Al Daghri, 2019 [28]	25OHD: enzyme linked immunosorbent assay	Tablet: 30.8 (9.3) Milk: 31.8 (8.1) C: 29.8 (10.3) (*p =* 0.46)	Tablet: 41.5 (14.4) Milk: 38.1 (11.9) C: 31.9 (13.8) (*p <* 0.001) (*p =* 0.73 between tablet and milk; <0.001 between tablet and C; *p <* 0.001 between milk and control)	SBP: Tablet: 117.3 (14.4) Milk: 117.9 (14.4) C: 120.9 (14.9) (*p =* 0.75)	SBP: Tablet: 112.3 (12.3) Milk: 115.3 (16.1) C: 117.1 (14.9) (*p =* 0.005) (*p =* 0.44 between tablet and milk; *p =* 0.004 between tablet and C; *p =* 0.19 between milk and C)	Significant decrease in SBP in all groups. Between-group significant decrease in favor of the tablet group (*p =* 0.005)
	SBP and DBP: average of 2 measures by a conventional mercurial sphygmomanometer taken after a 30-minute rest			DBP: Tablet: 71.9 (11.9) Milk: 73.3 (15.7) C: 72.5 (11.6) (*p =* 0.50)	DBP: Tablet: 69 (11.4) Milk: 75.6 (15.7) C: 68.1 (11.1) (*p <* 0.001) (*p <* 0.001 between tablet and milk; *p =* 0.94 between tablet and C; *p <* 0.001 between milk and C)	Significant decrease in DBP in the tablet and control groups. Between-group significant improvement in favor of control (*p <* 0.001)
	Elevated BP: ≥90th percentile for age, sex and height			Elevated BP: Tablet: 38.9% Milk: 40.7% C: 34.9%	Elevated BP: Tablet: 25.0% (*p <* 0.001) Milk: 44.4% (NS) C: 24.7% (*p <* 0.05)	Significant reduction in elevated BP in the tablet and control groups (*p <* 0.05)
Dong, 2010 [29]	Plasma 25OHD: enzyme immunoassay	I: 33.1 (8.7) C: 34.0 (10.6) (*p =* 0.76)	I: 85.7 (30.1) C: 59.8 (18.2) (*p <* 0.001)	SBPI: 111.3 (10.4) C: 114.9 (7.8) (*p =* 0.20)	NR	NS change in SBP or DBP over time in the control or intervention groups (*p >* 0.05)
	SBP and DBP: average of 3 measures, 2 minutes apart, by a vital signs monitor after a 5-minute rest			DBP I:68.2 (12.3) C: 69.4 (6.5) (*p =* 0.17)		
Hauger, 2018 [32]	Serum 25OHD: liquid chromatography-tandem mass spectrometry	I1: 56.9 (12.7) I2: 58.1 (13.5) C: 55.2 (10.8)	I1: 61.8 (10.6) I2: 75.8 (11.5) C: 31.1 (7.5) (*p <* 0.001)	SBP: I1: 95.7 (4.6) I2: 96.4 (4.4) C: 97.1 (5.5)	SBP: I1: 95.6 (4.4) I2: 96.9 (4.4) C: 96.6 (4.2) (NS)	NS change in SBP or DBP when adjusted for baseline value of the outcome
						Marginally higher DBP of 1.4 mmHg (95% CI: −0.0,2.8; *p =* 0.05) in I1 compared with I2
	SBP and DBP: average of 2 out of 3 readings, 10 minutes apart, by an automated monitor in a supine position			DBP: I1: 58.8 (4.1) I2: 59.9 (4.6) C: 59.2 (4.1)	DBP: I1: 58.5 (4.5) I2: 60.5 (3.8) C: 59.9 (3.3) (NS)	Marginally lower DBP of −1.2mmHg (95% CI: −2.7, −0.0; *p =* 0.05) in I1 compared with C, which was not observed with I2
Kelishadi, 2014 [30]	Serum 25OHD: chemiluminescent immunoassay method	I: 45.60 (5.09) C: 44.7 (5.66) (*p =* 0.48)	I: 79.89 (5.34) C: 47.59 (5.01) (*p =* 0.02)	I: 134.01 (5.89) C: 136.61 (6.08) (*p =* 0.53)	I: 131.47 (4.69) C: 135.26 (4.52) (*p =* 0.07)	NS change in mean arterial pressure in intra-group and inter-group comparisons
	Mean arterial pressure: ((SBP − DBP)/3) + DBP; with SBP and DBP measured using standard protocol with calibrated instruments (procedure not detailed)					
Smith, 2018 [31]	Serum 25OHD: liquid chromatography-tandem mass spectrometry	I1: 49.2 (12) I2: 51.7 (13.4) C: 46.9 (11.4)	I1: 56.6 (12.4) I2: 63.9 (10.6) C: 30.7 (8.6) (*p <* 0.001 for all groups, from baseline to endline)	SBP: I1: 124 (13) I2:122 (10) C: 121 (10)	SBP: I1: 123 (11) I2: 121 (8) C: 123 (14) (NS)	NS change in SBP or DBP
	SBP and DBP: average of 3 readings, 1 min apart, by an automated BP monitor on the non-dominant arm in an upright position with the arm supported			DBP: I1: 69 (10) I2: 67 (7) C: 67 (8)	DBP: I1: 71 (11) I2: 67 (6) C: 68 (10) (NS)	

SD: standard deviation; 25OHD: 25-hydroxyvitamin D; BP: blood pressure; SBP: systolic blood pressure; DBP: diastolic blood pressure; C: control; I: intervention; NR: not reported; NS: not significant.

## Supplementary Materials

The following are available online at https://www.mdpi.com/2072-6643/12/4/1163/s1.
