# Supplementary material for: Vitamin D Supplementation and Blood Pressure in Children and Adolescents: A Systematic Review and Meta-Analysis
Source: Nutrients. 2020 Apr 22;12(4):1163. doi: 10.3390/nu12041163 (PMC7230389; doi:10.3390/nu12041163)
Supplement: Supplementary file 1 [file nutrients-12-01163-s001.pdf]

Supplement 1: Search Strategy

MEDLINE

Database: Ovid MEDLINE(R) ALL <1946 to January 17, 2020>

Search Strategy:

----

1 exp Vitamin D/ (57819)

2 (Cholecalciferol* or calciol or HYDROXYCHOLECALCIFEROL* or hydroxyvitamins d or CALCIFEDIOL or 25 hydroxyvitamin d3 or 25-hydroxycholecalciferol or calcidiol or DIHYDROXYCHOLECALCIFEROL* or dihydroxyvitamins d or CALCITRIOL or 1 alpha,25 dihydroxyvitamin d3 or 1 alpha,25-dihydroxycholecalciferol or 1,25-dihydroxyvitamin d3 or ergocalciferol* or Dihydrotachysterol or 25-Hydroxyvitamin D 2).mp. [mp=title, abstract, original title, name of substance word, subject heading word, floating sub-heading word, keyword heading word, organism supplementary concept word, protocol supplementary concept word, rare disease supplementary concept word, unique identifier, synonyms] (38570)

3 (vitamin adj (d or d2 or d3 or d-2 or d-3)).mp. [mp=title, abstract, original title, name of substance word, subject heading word, floating sub-heading word, keyword heading word, organism supplementary concept word, protocol supplementary concept word, rare disease supplementary concept word, unique identifier, synonyms] (75439)

4 Vitamin D Deficiency/ (15265)

5 1 or 2 or 3 or 4 (87829)

6 exp Hypertension/ (250658)

7 hypertensi*.mp. (499579)

8 ((increase or elevat* or high*) adj2 blood adj1 pressure).mp. [mp=title, abstract, original title, name of substance word, subject heading word, floating sub-heading word, keyword heading word, organism supplementary concept word, protocol supplementary concept word, rare disease supplementary concept word, unique identifier, synonyms] (38782)

9 ((systolic or diastolic or arterial) adj3 pressure).mp. [mp=title, abstract, original title, name of substance word, subject heading word, floating sub-heading word, keyword heading word, organism supplementary concept word, protocol supplementary concept word, rare disease supplementary concept word, unique identifier, synonyms] (179307)

10 7 or 8 or 9 (627657)

11 adolescent/ or exp child/ (2958419)

12 (child or children or childhood or youth or teenage* or p?ediatric*).mp. [mp=title, abstract, original title, name of substance word, subject heading word, floating sub-heading word, keyword heading word, organism supplementary concept word, protocol supplementary concept word, rare disease supplementary concept word, unique identifier, synonyms] (2477749)

13 (adolescen* or (school adj age) or youngster*).mp. [mp=title, abstract, original title, name of substance word, subject heading word, floating sub-heading word, keyword heading word, organism supplementary concept word, protocol supplementary concept word, rare disease supplementary concept word, unique identifier, synonyms] (2066781)

14 11 or 12 or 13 (3499936)

15 5 and 10 and 14 (324)

16 5 and 10 (2408)

17 limit 16 to "all child (0 to 18 years)" (259)

18 15 or 17 (349)

19 (pediatric? or child or children or adolescen* or youth).jw. (542548)

20 16 and 19 (58)

21 18 or 20 (353)

EMBASE

Database: Embase Classic <1947 to 1973>, Embase <1974 to 2020 January 17>
Search Strategy:
----
1 exp vitamin D/ (140645)
2 exp vitamin D deficiency/ (28964)
3 (Cholecalciferol* or calciol or HYDROXYCHOLECALCIFEROL* or hydroxyvitamins d or CALCIFEDIOL or 25 hydroxyvitamin d3 or 25-hydroxycholecalciferol or calcidiol or DIHYDROXYCHOLECALCIFEROL* or dihydroxyvitamins d or CALCITRIOL or 1 alpha,25 dihydroxyvitamin d3 or 1 alpha,25-dihydroxycholecalciferol or 1,25-dihydroxyvitamin d3 or ergocalciferol* or Dihydrotachysterol or 25-Hydroxyvitamin D 2).mp. (52332)
4 (vitamin adj (d or d2 or d3 or d-2 or d-3)).mp. (130950)
5 1 or 2 or 3 or 4 (162755)
6 exp hypertension/ (733633)
7 hypertensi*.mp. (938420)
8 ((increase or elevat* or high*) adj2 blood adj1 pressure).mp. (58276)
9 ((systolic or diastolic or arterial) adj3 pressure).mp. (355528)
10 6 or 7 or 8 or 9 (1221108)
11 child/ (1769502)
12 exp adolescent/ (1484727)
13 (child or children or childhood or youth or teenage* or p?ediatric*).mp. (2867838)
14 (adolescen* or (school adj age) or youngster*).mp. (1596691)
15 11 or 12 or 13 or 14 (3542372)
16 5 and 10 and 15 (1398)
17 5 and 10 (10579)
18 limit 17 to child <unspecified age> (646)
19 16 or 18 (1398)

CINAHL

#	Query	Limiters/Expanders	Results
S17	S14 OR S16	Expanders - Apply equivalent subjects<br>Search modes - Boolean/Phrase	83
S16	S5 AND S9	Expanders - Apply equivalent subjects<br>Narrow by SubjectAge: - all child<br>Search modes - Boolean/Phrase	71
S15	S5 AND S9	Expanders - Apply equivalent subjects<br>Search modes - Boolean/Phrase	463
S14	S5 AND S9 AND S13	Expanders - Apply equivalent subjects<br>Search modes - Boolean/Phrase	83
S13	S10 OR S11 OR S12	Expanders - Apply equivalent subjects<br>Search modes - Boolean/Phrase	961,298
S12	Ti (youngster or school age or adolescen* or paediatric* or pediatric* or teenage* or youth or child*) or ab (youngster or school age or adolescen* or paediatric* or pediatric* or teenage* or youth or child*)	Expanders - Apply equivalent subjects<br>Search modes - Boolean/Phrase	398,135
S11	(MH "Adolescence+")	Expanders - Apply equivalent subjects<br>Search modes - Boolean/Phrase	477,443
S10	(MH "Child+")	Expanders - Apply equivalent subjects<br>Search modes - Boolean/Phrase	596,921
S9	S6 OR S7 OR S8	Expanders - Apply equivalent subjects<br>Search modes - Boolean/Phrase	81,207
S8	ti (high or elevated or increase* or systolic or diastolic or arterial) n3 pressure* or ab (high or elevated or increase* or systolic or diastolic or arterial) n3 pressure*	Expanders - Apply equivalent subjects<br>Search modes - Boolean/Phrase	5,573
S7	ti hypertensi* or ab hypertensi*	Expanders - Apply equivalent subjects<br>Search modes - Boolean/Phrase	35,745
S6	(MH "Hypertension+")	Expanders - Apply equivalent subjects<br>Search modes - Boolean/Phrase	69,915
S5	S1 OR S2 OR S3 OR S4	Expanders - Apply equivalent subjects<br>Search modes - Boolean/Phrase	20,348
S4	Ti (vitamin d or vitamin d2 or vitamin d3 or vitamin d 3 or vitamin d 2) or ab (vitamin d or vitamin d2 or vitamin d3 or vitamin d 3 or vitamin d 2)	Expanders - Apply equivalent subjects<br>Search modes - Boolean/Phrase	10,864
S3	Ti (alpha,25-dihydroxycholecalciferol or ergocalciferol* or Dihydrotachysterol or	Expanders - Apply equivalent subjects<br>Search modes - Boolean/Phrase	101

	dihydroxyvitamin d or dihydroxyvitamins d or 1 alpha,25 dihydroxyvitamin d3 or 1 alpha,25-dihydroxycholecalciferol or 1,25-dihydroxyvitamin d3 or 25-Hydroxyvitamin D 2) or ab (alpha,25-dihydroxycholecalciferol or ergocalciferol* or Dihydrotachysterol or dihydroxyvitamin d or dihydroxyvitamins d or 1 alpha,25 dihydroxyvitamin d3 or 1 alpha,25-dihydroxycholecalciferol or 1,25-dihydroxyvitamin d3 or 25-Hydroxyvitamin D 2)		
S2	Ti (Cholecalciferol* or calciol or HYDROXYCHOLECALCIFEROL* or CALCIFEDIOL or 25-hydroxycholecalciferol or calcidiol or DIHYDROXYCHOLECALCIFEROL* or CALCITRIOL) or ab (Cholecalciferol* or calciol or HYDROXYCHOLECALCIFEROL* or CALCIFEDIOL or 25-hydroxycholecalciferol or calcidiol or DIHYDROXYCHOLECALCIFEROL* or CALCITRIOL)	Expanders - Apply equivalent subjects<br>Search modes - Boolean/Phrase	360
S1	(MH "Vitamin D+") OR (MH "Vitamin D Deficiency+")	Expanders - Apply equivalent subjects<br>Search modes - Boolean/Phrase	18,743

PUBMED

Search	Query	Items found
#29	Search (#7 and #17 and #28)	3648
#28	Search (#18 or #19 or #20 or #21 or #22 or #23 or #24 or #25 or #26 or #27)	3454758
#27	Search youngster*[tiab]	2476
#26	Search school age[tiab]	13013
#25	Search adolescen*[tiab]	286605
#24	Search paediatric*[tiab]	64591
#23	Search pediatric*[tiab]	289152
#22	Search teenage*[tiab]	20898
#21	Search youth[tiab]	68591
#20	Search ((child[tiab] or children[tiab] or childhood[tiab]))	1333474
#19	Search child[mesh]	1872154
#18	Search adolescent[mesh]	1983762
#17	Search (# 8 or #9 or #10 or #11 or #12 or #13 or #14 or #15 or #16)	4646659
#16	Search (hypertension[tiab] or hypertensive[tiab] or hypertensives[tiab])	426502
#15	Search arterial pressure[tiab]	58738
#14	Search diastolic pressure[tiab]	13643
#13	Search systolic pressure[tiab]	13954
#12	Search increase blood pressure*[tiab]	760
#11	Search elevated blood pressure[tiab]	6289
#10	Search ((systolic blood pressure*[tiab] or diastolic blood pressure*[tiab] or arterial blood pressure*[tiab]))	98847
#9	Search high blood pressure*[tiab]	14672
#8	Search hypertension[mesh]	250320
#7	Search (#1 or #2 or #3 or #4 or #5 or #6)	87699
#6	Search (vitamin d[tiab] or vitamin d2[tiab] or vitamin d3[tiab] or vitamin d 3[tiab] or vitamin d 2[tiab])	66554
#5	Search (dihydroxyvitamin d[tw] or dihydroxyvitamins d[tw] or 1 alpha,25 dihydroxyvitamin d3[tw] or 1 alpha,25-dihydroxycholecalciferol[tw] or 1,25-dihydroxyvitamin d3[tw] or 25-Hydroxyvitamin D 2[tw])	12196
#4	Search 25 hydroxyvitamin d3 [tw]	2989
#3	Search (Cholecalciferol*[tw] or calciol[tw] or HYDROXYCHOLECALCIFEROL*[tw] or CALCIFEDIOL[tw] or 25-hydroxycholecalciferol[tw] or calcidiol[tw] or DIHYDROXYCHOLECALCIFEROL*[tw] or CALCITRIOL[tw] or alpha,25-dihydroxycholecalciferol[tw] or ergocalciferol*[tw] or Dihydrotachysterol[tw])	36629
#2	Search vitamin D deficiency [mesh:noexp]	15220
#1	Search vitamin D [mesh]	57729

Cochrane Library
#1 MeSH descriptor: [Vitamin D] explode all trees 4778
#2 MeSH descriptor: [Vitamin D Deficiency] explode all trees 1269
#3 (Cholecalciferol* or calciol or HYDROXYCHOLECALCIFEROL* or CALCIFEDIOL):ti,ab,kw 3039
#4 (calcidiol or DIHYDROXYCHOLECALCIFEROL* or CALCITRIOL):ti,ab,kw 1897
#5 (ergocalciferol* or Dihydrotachysterol or dihydroxyvitamin d or dihydroxyvitamins d or 1 alpha,25 dihydroxyvitamin d3):ti,ab,kw 1826
#6 (vitamin d):ti,ab,kw 12995
#7 (vitamin d2 or vitamin d 3):ti,ab,kw 7528
#8 #1 or #2 or #3 or #4 or #5 or #6 or #7 14586
#9 MeSH descriptor: [Hypertension] explode all trees 17001
#10 (hypertensi* or (high or elevated or increase* or systolic or diastolic or arterial) near/3 pressure*):ti,ab,kw 568810
#11 #9 or #10 568810
#12 MeSH descriptor: [Child] explode all trees 1209
#13 MeSH descriptor: [Adolescent] explode all trees 102003
#14 (youngster or school age or adolescen* or paediatric* or pediatric* or teenage* or youth or child*):ti,ab,kw 243608
#15 #12 or #13 or #14243608
#16 #8 and #11 and #15 1559
1525 Trials matching "#16 - #8 and #11 and #15"

ClinicalTrial.gov
Hypertension | Vitamin D | Child
Applied filer: Child (birth-17)

Supplement Figure S1: Sensitivity analyses

Study or Subgroup	Post Mean	Post SD	Post Total	Pre Mean	Pre SD	Pre Total	Weight	Mean Difference IV, Random, 95% CI
Ashraf 2011	118	15	14	123	14	14	1.4%	-5.00 [-15.75, 5.75]
Khayyatzadeh 2018	97.16	14.5	942	96.69	14.2	942	98.6%	0.47 [-0.83, 1.77]
**Total (95% CI)**			**956**			**956**	**100.0%**	**0.39 [-0.90, 1.68]**

Heterogeneity: Tau² = 0.00; Chi² = 0.98, df = 1 (P = 0.32); I² = 0%
Test for overall effect: Z = 0.60 (P = 0.55)

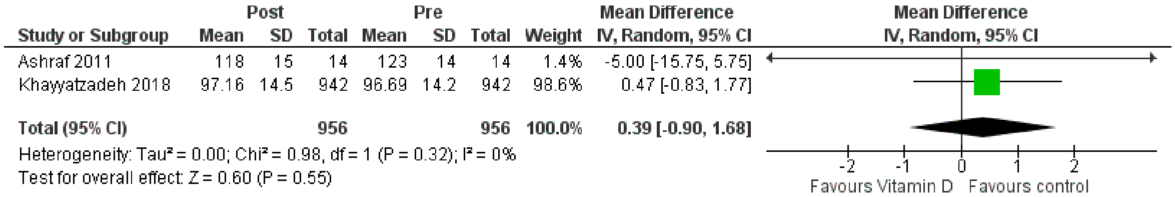

Figure a. Forest plot of sensitivity analysis of mean differences in SBP (in mmHg) before and after subjects receiving vitamin D supplementation (excluding vitamin D sufficient patients from Khayyatzadeh, 2018).

Study or Subgroup	Post Mean	Post SD	Post Total	Pre Mean	Pre SD	Pre Total	Weight	Mean Difference IV, Random, 95% CI
Ashraf 2011	64	8	14	65	9	14	3.3%	-1.00 [-7.31, 5.31]
Khayyatzadeh 2018	60.71	12.88	942	62.61	13.04	942	96.7%	-1.90 [-3.07, -0.73]
**Total (95% CI)**			**956**			**956**	**100.0%**	**-1.87 [-3.02, -0.72]**

Heterogeneity: Tau² = 0.00; Chi² = 0.08, df = 1 (P = 0.78); I² = 0%
Test for overall effect: Z = 3.18 (P = 0.001)

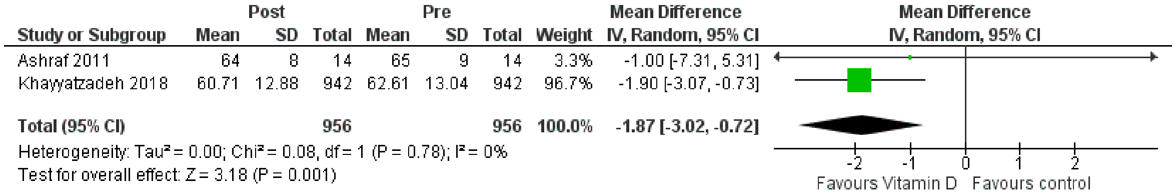

Figure b. Forest plot of sensitivity analysis of mean differences in DBP (in mmHg) before and after subjects receiving vitamin D supplementation (excluding vitamin D sufficient patients from Khayyatzadeh, 2018).

Study or Subgroup	Vitamin D supplementation Mean	SD	Total	Control Mean	SD	Total	Weight	Mean Difference IV, Random, 95% CI
Hauger 2018	96.2	4.4	87	96.6	4.2	43	90.2%	-0.40 [-1.96, 1.16]
Smith 2018	122	9.67	75	123	14	43	9.8%	-1.00 [-5.72, 3.72]
**Total (95% CI)**			**162**			**86**	**100.0%**	**-0.46 [-1.94, 1.02]**

Heterogeneity: Tau² = 0.00; Chi² = 0.06, df = 1 (P = 0.81); I² = 0%
Test for overall effect: Z = 0.61 (P = 0.54)

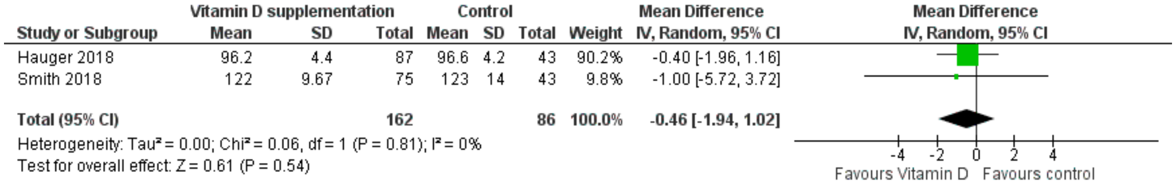

Figure c. Forest plot of sensitivity analysis of mean differences in SBP (in mmHg) between subjects receiving vitamin D supplementation compared with their comparators (excluding Al Daghri, 2019 as the source of heterogeneity).

Study or Subgroup	Vitamin D supplementation Mean	SD	Total	Control Mean	SD	Total	Weight	Mean Difference IV, Random, 95% CI
Hauger 2018	59.49	4.26	87	59.9	3.3	43	88.1%	-0.41 [-1.74, 0.92]
Smith 2018	69.08	9.12	75	68	10	43	11.9%	1.08 [-2.55, 4.71]
**Total (95% CI)**			**162**			**86**	**100.0%**	**-0.23 [-1.48, 1.02]**

Heterogeneity: Tau² = 0.00; Chi² = 0.57, df = 1 (P = 0.45); I² = 0%
Test for overall effect: Z = 0.37 (P = 0.71)

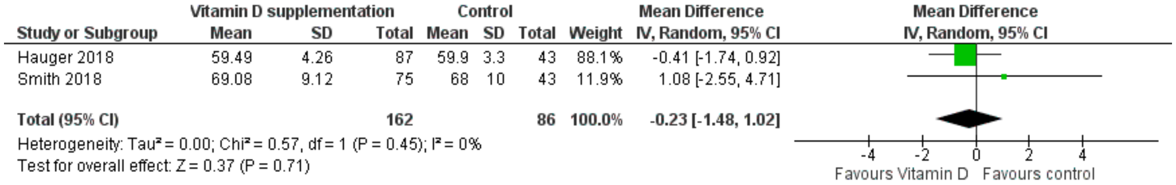

Figure d. Forest plot of sensitivity analysis of mean differences in SBP (in mmHg) between subjects receiving vitamin D supplementation compared with their comparators (excluding Al Daghri, 2019 which included only vitamin D deficient patients)

Figure S1. Meta-analysis of the sensitivity analyses of the effects of vitamin D supplementation on SBP and DBP. Mean differences for each study are represented by squares, and 95% CIs are represented by the lines through the squares. The pooled mean differences are represented by diamonds. Between-study heterogeneity was assessed with the use of the I2 statistic. SBP: Systolic blood pressure; DBP: Diastolic blood pressure.

Supplement: GRADEing of the Meta-analysis

Table S1: Quality of evidence of NRS and RCTs

NRS	Certainty assessment					№ of patients		Effect	Certainty
**№ of studies**	**Study design**	**Risk of bias**	**Inconsistency**	**Indirectness**	**Imprecision**	**Pre-supplementation**	**Post-supplementation**	**Absolute (95% CI)**	
**Systolic blood pressure**									
2	Observational studies	Not serious	Not serious	Not serious	Serious [b]	1002	1002	MD **0.33 higher (0.93 lower to 1.58 higher)**	⊕◯◯◯ VERY LOW
**Diastolic blood pressure**									
2	Observational studies	Not serious	Not serious	Not serious	Not serious	1002	1002	MD **2.26 lower (3.4 lower to 1.12 lower)**	⊕⊕◯◯ LOW
**RCTs**	**Certainty assessment**					**№ of patients**		**Effect**	**Certainty**
**№ of studies**	**Study design**	**Risk of bias**	**Inconsistency**	**Indirectness**	**Imprecision**	**Supplementation**	**No supplementation**	**Absolute (95% CI)**	
**Systolic blood pressure**									
3	Randomized trials	Not serious [a]	Serious [c]	Not serious	Serious [b]	328	266	MD **2.04 lower (5.12 lower to 1.04 higher)**	⊕⊕◯◯ LOW
**Diastolic blood pressure**									
3	Randomized trials	Not serious [a]	Sot serious	Not serious	Serious [b]	328	266	MD **0.01 higher (1.09 lower to 1.12 higher)**	⊕⊕⊕◯ MODERATE

CI: Confidence interval; **MD:** Mean difference; **NRS:** Non-randomized human interventions study; **RCT:** Randomized controlled trial

Explanations

a. Al Daghri (2019) has some risk of bias with respect to blinding of participants, personnel and outcome assessors and to other bias. It contributed to only 21% of the weight.

b. Downgraded by one level due to serious imprecision. Confidence interval crosses the line of no effect.

c. Downgraded by one level due to serious inconsistency. Unexplained heterogeneity I2=71%.

Table S2: Sensitivity analyses of the quality of evidence of NRS and RCTs

NRS	Certainty assessment					№ of patients		Effect	Certainty
**№ of studies**	**Study design**	**Risk of bias**	**Inconsistency**	**Indirectness**	**Imprecision**	**Vitamin D supplementation**	**No supplementation**	**Absolute (95% CI)**	
**Systolic blood pressure (excluding vitamin D sufficient patients from Khayyatzadeh, 2018)**									
2	Observational studies	Not serious	Not serious	Not serious	Serious [a]	956	956	MD **0.39 higher (0.9 lower to 1.68 higher)**	⊕◯◯◯ VERY LOW
**Diastolic blood pressure (excluding vitamin D sufficient patients from Khayyatzadeh, 2018)**									
2	Observational studies	Not serious	Not serious	Not serious	Not serious	956	956	MD **1.87 lower (3.02 lower to 0.72 lower)**	⊕⊕◯◯ LOW
**RCTs**	**Certainty assessment**					**№ of patients**		**Effect**	**Certainty**
**№ of studies**	**Study design**	**Risk of bias**	**Inconsistency**	**Indirectness**	**Imprecision**	**Vitamin D supplementation**	**No supplementation**	**Absolute (95% CI)**	
**Systolic blood pressure (excluding Al Daghri, 2019 as the source of heterogeneity)**									
2	Randomized trials	Not serious	Not serious	Not serious	Serious [a]	162	86	MD **0.46 lower (1.94 lower to 1.02 higher)**	⊕⊕⊕◯ MODERATE
**Diastolic blood pressure (excluding Al Daghri, 2019 which included only vitamin D deficient patients)**									
2	Randomized trials	Not serious	Not serious	Not serious	Serious [a]	162	86	MD **0.23 lower (1.48 lower to 1.02 higher)**	⊕⊕⊕◯ MODERATE

CI: Confidence interval**; MD:** Mean difference**; NRS:** Non-randomized human interventions study**; RCT:** Randomized controlled trial

Explanations

a. Downgraded by one level due to serious imprecision. Confidence interval crosses the line of no effect.
